# COVID-19-Induced Left Sciatic Neuropathy Requiring Prolonged Physical Medicine and Rehabilitation

**DOI:** 10.7759/cureus.15803

**Published:** 2021-06-21

**Authors:** Sameer Acharya, Melissa Thibault, Janette Lee, Omar Taha, Andrew J Morpurgo, Binay K Kshetree, Kushal Regmi

**Affiliations:** 1 Internal Medicine, Cayuga Medical Center, Ithaca, USA; 2 Physical Medicine and Rehabilitation, Cayuga Medical Center, Ithaca, USA; 3 Pulmonary and Critical Care Medicine, Cayuga Medical Center, Ithaca, USA; 4 Internal Medicine, Chitwan Medical College, Chitwan, NPL

**Keywords:** covid 19, mono-neuropathy, sciatic, neurological effects of coronavirus, physical medicine and rehabilitation

## Abstract

A growing number of case reports and series have described a wide spectrum of neurological manifestations of COVID-19 disease including encephalopathy, cerebrovascular disease, and Guillain-Barre syndrome (GBS). However, peripheral neuropathy associated with COVID-19 disease has been uncommonly reported. Here, we describe a young patient with a COVID-19 infection who developed unilateral sciatic neuropathy during the course of treatment requiring prolonged physical medicine and rehabilitation stay. She was treated in the intensive care unit (ICU) for hypoxic respiratory failure for 22 days total, during which she was intubated, sedated, and paralyzed for 14 days. She received dexamethasone, convalescent plasma, and remdesivir for COVID-19; she also received ceftriaxone and azithromycin for possible superimposed bacterial pneumonia. The hypoxic respiratory failure was improved progressively, and she was extubated. On day 17 of ICU stay, she reported numbness and weakness in left leg and had 0/5 motor strength at the left ankle in all directions. She was able to move left hip and knee and had decreased sensation to light touch and pain from the level of the left knee to the toes. Imaging of the brain and spine showed no obvious findings that would explain the neurological symptoms. On electromyography (EMG), there was acute denervation in the left tibialis anterior muscle. She required prolonged physical medicine and rehabilitation care, greater than 60 days during which she had some improvement in sensation, but remained without ankle movement for two more months. This could be a rare manifestation of COVID-19-induced sciatic mono-neuropathy given her symptoms, EMG reports, clinical exam, and normal imaging studies.

## Introduction

The COVID-19 pandemic has been dreadful. In addition to hypoxic respiratory failure, there are multiple other organ systems afflicted by the virus. Not surprisingly, COVID-19-induced neurological symptoms are also emerging [1]. Although COVID-19-induced anosmia and ageusia have been reported from the start of the pandemic, severe neurological manifestations like Guillain-Barre syndrome (GBS) and large vessel strokes are being recognized many more frequently [2, 3]. A growing number of case reports and series describe a wide spectrum of neurological manifestations [2, 4]. However, COVID-19-induced peripheral neuropathy has been rarely reported [4, 5]. With the course of time, there have been an increasing concerns about COVID-19-induced neuropathy whose pathophysiology is yet to be understood [6]. Here, we present a case of a 34-year-old female with COVID-19-induced unilateral sciatic neuropathy requiring prolonged physical medicine and rehabilitation stay.

## Case presentation

A 34-year-old female with a past medical history of hypertension, morbid obesity, and obstructive sleep apnea presented to the emergency room with worsening shortness of breath. She had a positive COVID-19 polymerase chain reaction test, four days prior. She also had nasal congestion, sore throat, productive cough with yellow sputum, mild headache, and intermittent diarrhea. She denied fever, chest pain, loss of taste and smell. Her vital signs on ICU admission were: temperature 38.7°C, heart rate 83 bpm, respiratory rate 38 breaths per minute, oxygen saturation 96% on Vapotherm with FiO_2_ 100% 40 L/minute flow, and blood pressure 147/83 mmHg. Her body mass index was 67.5 kg/m^2^. Her oxygenation progressively worsened. She was in visible respiratory distress. She was using accessory muscles and had coarse distant breath sounds bilaterally. Her abdomen was obese with skin breakdown between abdominal fat pads. Lower extremities were edematous with skin breakdown and venous stasis changes. The neurological exam was unremarkable with normal strength throughout. She required intubation shortly after admission. Her complete blood count and complete metabolic panel were normal; she did have elevated lactate dehydrogenase 521 U/L, d-dimers 529 ng/ml and C-reactive protein of 233.81 mg/L, and an international normalized ratio of 1.38. Arterial blood gas showed respiratory acidosis with metabolic compensation. Computed tomography scan of chest revealed bilateral ground-glass pulmonary infiltrates with areas of atelectasis and consolidation consistent with COVID-19 pneumonia. She was treated in ICU for hypoxic respiratory failure for 22 days total, with 14 days of intubation with lung-protective mechanical ventilation, temporary paralysis, and sedation. She received dexamethasone, convalescent plasma, and remdesivir. She was also treated with ceftriaxone and azithromycin for possible superimposed bacterial pneumonia. Her hypoxia and respiratory failure progressively improved and she was extubated.

**Figure 1:**
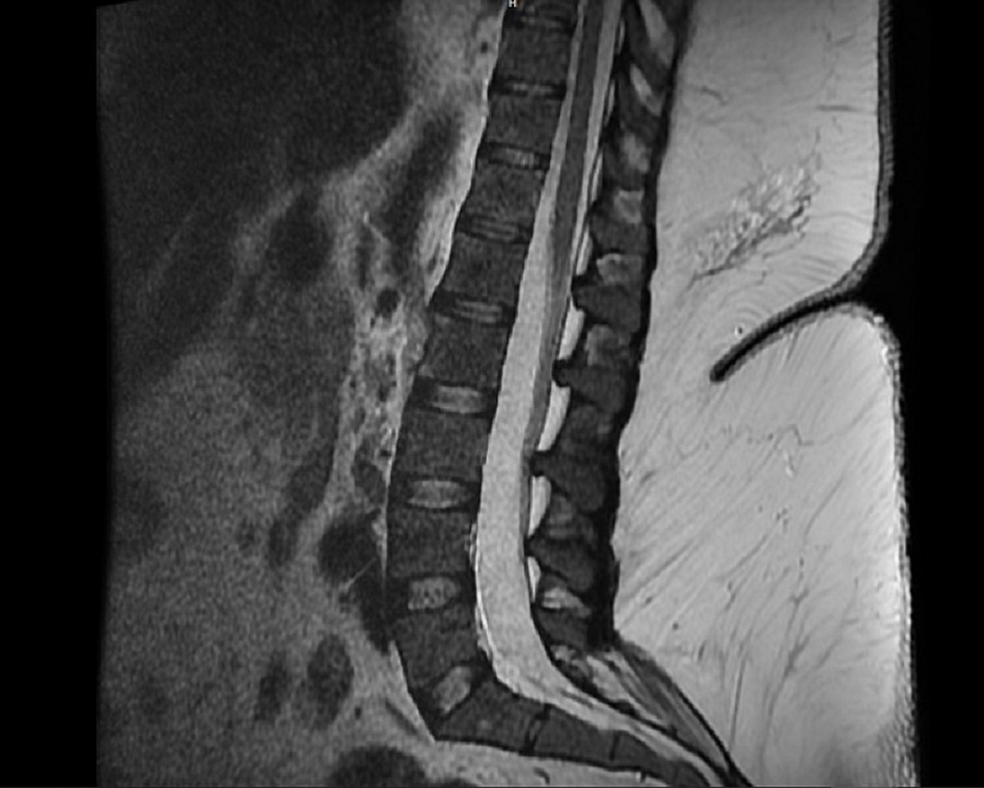
MRI Lumbosacral Spine MRI lumbosacral spine showing normal intervertebral disc spaces, vertebral bodies, and spinal cord.

Unfortunately, during the course of treatment, on ICU day 17, she reported worsening numbness and weakness in the left leg. She had 0/5 motor strength below the left knee and ankle joint and was only able to flex at the hip. She also had decreased sensation to light touch and pain from the left knee to toes. Magnetic resonance imaging (MRI) of the brain and lumbar spine (Figure 1) and computed tomography scan of the cervical, thoracic and lumbar spine showed no obvious findings that would explain her acute unilateral sciatic neuropathy. On electromyography (EMG), there was acute and chronic denervation in the left Tibialis anterior muscle. No tibial muscles were sampled due to concern for skin infection.

**Figure 2:**
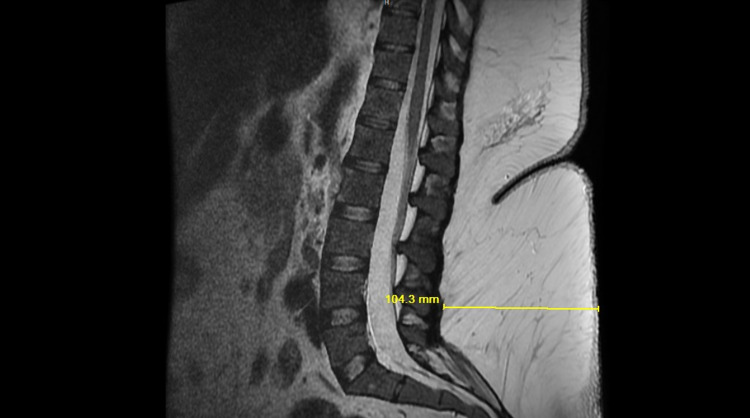
MRI Lumbosacral Spine MRI lumbosacral spine showing 104.3 mm depth of nerve roots through subcutaneous tissues from the skin.

As seen in MRI (Figure 2) there is more than 10 cm depth from the skin to the spine. Our longest EMG needle of 7.6 cm would not reach the paraspinal muscles. She required prolonged care in a physical medicine and rehabilitation unit for more than 60 days. She was provided comprehensive physical and occupational therapies and trainings which include trunk and lower extremity strengthening exercises, functional training of bed mobility, dynamic sitting balance, manual stretching of feet and legs and standing weight bearing with hoyer. Her ambulation was supported with multi-podus boots. Her neuropathic symptoms persisted for more than four months and completely recovered thereafter.

## Discussion

Though, the transmission of the severe acute respiratory syndrome coronavirus 2 (SARS-COV2 virus) among human beings has been believed to be via close contact (droplets) or distant (aerosol particles), contaminated surfaces, or fecal transmission [7], the exact mechanism of nervous system involvement is still unknown [6]. However, the possible routes of transmission could be retrograde neuronal transport across infected neurons, entry via the olfactory nerve, infection of the vascular endothelium, leucocytes migration across the blood-brain barrier, or via angiotensin-converting enzyme-2 receptors found on neurons and glial cells [8]. SARS-COV2-induced peripheral neuropathy could also have been contributed by the inflammatory or immunological response of neuronal cells reacting to the virus particles.

We are reporting this case as a rare manifestation of COVID-19-induced sciatic mono-neuropathy. Morbid obesity and prolonged ICU stay in the setting of COVID-19 have contributed to the presentation of our case, the latter being the most important cause. Critical care myopathy or obesity would not present with the features of unilateral isolated nerve injury [9, 10]. In our case, the imaging studies have also ruled out the spinal compressive causes that would foster these neurological symptoms. Involvement of other infectious or autoimmune etiologies is extremely unlikely in unilateral isolated large fiber mono-neuropathy and the absence of other systemic symptoms. Given her improving condition and large body habitus, we did not opt for a nerve biopsy. Though our EMG reports are suboptimal given her huge subcutaneous tissues but have shown some evidence of neuropathy that is not explained by any other causes. From this case report, the authors want to highlight the need for further studies to determine the exact mechanism of central or peripheral nervous system invasion by the virus and neurological complications of the COVID-19 disease.

## Conclusions

Though COVID-19 has been reported to be associated with neurologic manifestations, isolated large fiber mono-neuropathy has been rarely described. The authors want to highlight the importance of the early institution of physical rehabilitation that can help in mitigating the peripheral neuropathy complicated by the COVID-19 disease. With reports of neurologic disorders like GBS and large vessel strokes being a presenting feature or a complication of COVID-19 infections, the identification of even rarer neurologic sequel such as large fiber mono-neuropathies should not be overlooked, especially as it has long-term effects on recovery after an ICU discharge.
